# Evidence of a Link between Hepatitis E Virus Exposure and Glomerulonephritis Development

**DOI:** 10.3390/v15061379

**Published:** 2023-06-15

**Authors:** Mohamed A. El-Mokhtar, Ayat M. Kamel, Ehsan M. W. El-Sabaa, Sahar A. Mandour, Ahmed Shawkat Abdelmohsen, Abdelmajeed M. Moussa, Eman H. Salama, Sahar Aboulfotuh, Lobna Abdel-Wahid, Essam M. Abdel Aziz, Nashwa Mostafa A. Azoz, Ibrahim M. Sayed, Amal A. Elkhawaga

**Affiliations:** 1 Department of Medical Microbiology and Immunology, Faculty of Medicine, Assiut University, Assiut 71515, Egypt; 2 Microbiology and Immunology Department, Faculty of Pharmacy, Assiut University, Assiut 71515, Egypt; 3 Department of Microbiology and Immunology, Faculty of Pharmacy, Deraya University, Minia 11566, Egypt; 4 Department of Tropical Medicine and Gastroenterology, Faculty of Medicine, Assiut University, Assiut 71515, Egypt; 5 Department of Tropical Medicine and Gastroenterology, Faculty of Medicine, Aswan University, Aswan 81528, Egypt; 6 Department of Clinical Pathology, Faculty of Medicine, Sohag University, Sohag 82524, Egypt; 7 Gastroenterology and Hepatology Unit, Internal Medicine Department, Assiut University, Assiut 71515, Egypt; 8 Department of Internal Medicine, Nephrology Division, Faculty of Medicine, Assiut University, Assiut 71515, Egypt

**Keywords:** HEV, glomerulonephritis, seroprevalence, anti-HEV IgG, extrahepatic disorder

## Abstract

Viruses can trigger glomerulonephritis (GN) development. Hepatitis viruses, especially Hepatitis C virus and Hepatitis B viruses, are examples of the viruses that trigger GN initiation or progression. However, the proof of a correlation between GN and Hepatitis E virus infection is not clear. Some studies confirmed the development of GN during acute or chronic HEV infections, mainly caused by genotype 3. While others reported that there is no relation between HEV exposure and GN development. A recent study showed that a reduced glomerular filtration rate was developed in 16% of acute HEV genotype 1 (HEV-1) infections that returned to normal during recovery. HEV-1 is endemic in Egypt with a high seroprevalence among villagers and pregnant women. There is no available data about a link between HEV and GN in Egypt. Methods: GN patients (n = 43) and matched healthy subjects (n = 36) enrolled in Assiut University hospitals were included in this study. Blood samples were screened for hepatotropic pathogens. Tests for HEV markers such as HEV RNA and anti-HEV antibodies (IgM and IgG) were performed. Laboratory parameters were compared in HEV-seropositive and HEV-seronegative GN patients. Results: Anti-HEV IgG was detected in 26 (60.5%) out of 43 GN patients. HEV seroprevalence was significantly higher in GN than in healthy controls, suggesting that HEV exposure is a risk factor for GN development. None of the GN patients nor the healthy subjects were positive for anti-HEV IgM or HEV RNA. There was no significant difference between seropositive and seronegative GN patients in terms of age, gender, albumin, kidney function profiles, or liver transaminases. However, anti-HEV IgG positive GN patients had higher bilirubin levels than anti-HEV IgG negative GN patients. HEV-seropositive GN patients had a significantly elevated AST level compared to HEV-seropositive healthy subjects. Conclusion: exposure to HEV infection could be complicated by the development of GN.

## 1. Introduction

Glomerulonephritis (GN) is an inflammation in the glomeruli that leads to a decrease in the performance of the kidneys to excrete waste products. Viral infections can trigger the development of GN such as Hepatitis B virus (HBV), Hepatitis C virus (HCV), Human immunodeficiency virus (HIV), cytomegalovirus (CMV), and Epstein–Barr virus (EBV) [1]. Not only in serologically positive hepatitis patients, but GN is also developed in occult HBV- or HCV-infected patients; viral antigens such as HBs Ag, HBc Ag, and HCV Ag were recorded in kidney biopsies [2]. Additionally, viral particles were detected in the frozen renal tissues [2]. Regarding HEV infection, GN was also reported in HEV-infected patients, especially in patients with cryoglobulinemia [3,4,5,6]. Although the viral protein, nucleic acid was recorded in the urine of HEV-infected patients, suggesting renal involvement associated with HEV infection [7], the link between HEV infection with glomerulonephritis is not completely understood, especially with certain HEV genotypes.

HEV is an enterically transmitted virus belonging to the *Hepeviridae* family [8]. Mammalian HEV strains are recently categorized into the genus *Paslahepevirus* and the subfamily *Orthohepevirinae* [8]. Not all HEV isolates were reported with renal disorders and/or GN. HEV genotype 3 (HEV-3) is mainly reported with GN [9]. Several studies confirmed the possible association between HEV infection and the development of GN during acute and chronic phases of HEV infections [3,5,10]. However, Pischke et al. showed that there is no link between previous exposure to HEV-3 and the development of GN in general [11]. Regarding HEV genotype 1 (HEV-1), limited data were linked to renal disorders. A recent study showed that 16% of acute HEV-1 developed abnormal kidney function parameters during the acute phase of infection. However, those patients had not developed further renal complications, and the renal parameters improved at convalescence [12]. Few other case studies reported an association between HEV-1 infection and membranous glomerulonephritis, and/or cholemic nephrosis [13,14]. An in vitro study using primary proximal epithelial cells showed that HEV-1 could replicate efficiently in the renal epithelium inducing inflammatory responses and renal injury due to the crosstalk and link between IL-18 and the interferon-γ signaling pathway [15].

In Egypt, hepatotropic pathogens affect many populations. GN was recorded in Egyptians in association with HCV or HBV infection [16,17]. The prevalence of anti-HBC- antibodies was significantly higher in GN children (30%) in Egypt than in matched healthy controls [17]. Several studies in the last two decades revealed that HEV infection is endemic in Egypt [18,19]. A high rate of anti-HEV IgG seroprevalence is reported in rural communities, especially among villagers and pregnant women [18,19,20,21]. HEV-infected patients were either asymptomatic or developed the acute self-limiting disease [20,22,23,24]. The actual prevalence of HEV infection in Egypt is not known due to either silent infection and/or the infection not being documented or monitored. A recent study showed that HEV infection was recorded in acute hepatitis patients of unknown etiology, in coinfection with other hepatotropic pathogens such as adenovirus, hepatitis A virus (HAV), and *Coxiella burnetii* [25,26]. HEV-1 is the predominant isolate among Egyptians [22,27,28,29], and few cases were reported with HEV-3 [29,30]. However, the available data that could link HEV-1 exposure and/or infection and GN is missing.

Herein, we aimed to assess the relation of HEV exposure to the development of GN. We assessed HEV markers in GN patients and matched healthy subjects. We found that a significantly higher anti-HEV IgG was recorded in GN patients compared to healthy controls. We also compared HEV-seropositive and HEV-seronegative GN patients based on liver and kidney profiles. We believe that exposure to HEV could be linked directly or indirectly to GN development. To our knowledge, this is the first report to assess HEV markers in GN in regions endemic to HEV-1 infections.

## 2. Materials and Methods

### 2.1. Patients, HEV Diagnosis, and Flow Chart

This study included an analysis of GN patients (n = 43) admitted to outpatient clinics and the Nephrology department in Assiut University Hospitals, Egypt during the period from December 2020 to January 2022. Those patients had clinical manifestations of GN that included the presence of red blood cells in the urine, impaired kidney functions, proteinuria, nausea, vomiting, muscle cramps, edema in different body sites, and decreased urine elimination. Healthy subjects (n = 36) of similar age, sex-matched, and from the same geographical locations were enrolled in the study as controls. Blood samples were centrifuged at 1000–2000× *g* for 10 min. Plasma/sera samples were stored at −20 °C (for ELISA) or at −80 °C (for RNA) till the time of analysis. Viral markers were assessed using serological and molecular approaches. For molecular viral detection, viral nucleic acids (DNA or RNA) were extracted from patients’ sera samples using QIAamp MinElute Virus nucleic acid extraction kit (Qiagen, Hilden, Germany) according to the manufacturer’s instructions. For RT-qPCR, RNA is first converted into cDNA using a High-Capacity cDNA Reverse Transcription Kit (ThermoFisher Scientific, Waltham, MA, USA) according to the manufacturer’s instructions. HEV RNA was tested in these samples using primers targeting HEV ORF2/3 and TaqMan™ Fast Virus 1-Step Master Mix by RT-qPCR according to the manufacturer’s instructions, and HEV ORF2 was detected by nested PCR as described previously [25,28,29]. For HBV DNA, qPCR was performed using the artus HBV RG PCR kit (Qiagen, Germany) according to the manufacturer’s instructions. RT-qPCR was run for detection of HAV RNA using primers listed in Table 1. Detection and quantification of HCV RNA by RT-qPCR using the artus HCV RG RT-PCR kit according to the manufacturer’s instructions. CMV and adenovirus were also analyzed using PCR by detection of CMV DNA and adenovirus DNA using primers listed in Table 1.

Serological assessment of viral hepatitis markers was also performed. Analysis of anti-HEV IgM and anti-HEV IgG was conducted using indirect solid-phase enzyme ELISA assays (the abia, AB Diagnostic Systems GmbH, Berlin, Germany) according to the manufacturer’s instructions. The colour intensity is read at 450 nm or 450/620 nm and directly proportional to the concentration of HEV antibodies in the specimen. Hepatitis A virus immunoglobulin M was conducted using an anti-HAV IgM kit (CTK Biotech, Poway, CA, USA) according to the manufacturer’s instructions. Serological assays for HBV included the detection of anti-HBV core IgM and HBs Ag using commercial ELISA assays (Prechek Bio Inc., Taiwan) according to the manufacturer’s instructions. HCV was analyzed by assessment of anti-HCV antibodies using the fourth-generation HCV TRI-DOT test control (Atlas Link, Manassas, VA, USA) according to the manufacturer’s instructions. Other viruses were tested by the detection of antiviral antibodies using commercial ELISA assays such as anti-CMV antibodies (Serion ELISA classic, Würzburg, Germany), anti-EBV antibodies (Serion ELISA classic, Würzburg, Germany), and anti-parvovirus (Serion ELISA classic, Würzburg, Germany) according to the manufacturer’s instructions.

**Figure 1:**
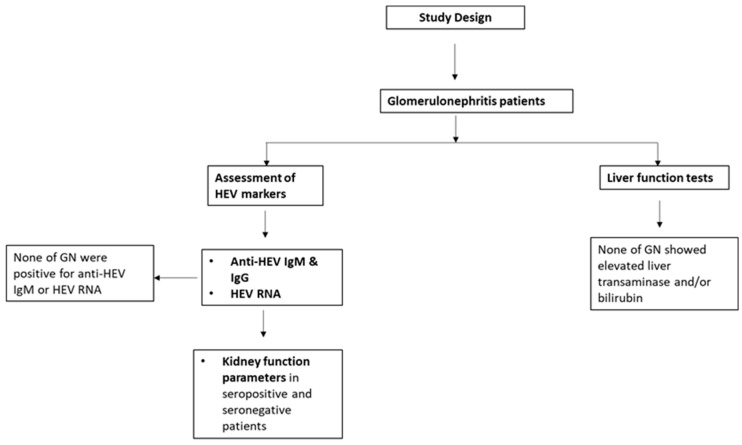
Flow chart and study design: GN patients were assessed for HEV markers (HEV RNA, anti-HEV IgM, and anti-HEV IgG) and LFTs (ALT, AST, and bilirubin). KFTs were analyzed in HEV seropositive and HEV seronegative patients.

Liver function and kidney functions were assessed in both groups using a clinical chemistry analyzer (Mindray BS-230). The flow chart of this study is presented in Figure 1.

### 2.2. Statistical Analysis

The data are expressed as the mean ± standard deviation (SD) or median and the range (minimum–maximum). They were compared using the parametric Student’s *t*-test, and/or the non-parametric Mann–Whitney U-test. All tests were two-tailed, and *p*-values were determined; *p* < 0.05 were considered significant. The analyses were performed using GraphPad Prism 9.

## 3. Results

### 3.1. High Seroprevalence of Anti-HEV IgG among GN Patients

In this study, we analyzed markers of hepatotropic pathogens among GN patients (n = 43). None of the enrolled patients were infected with viruses that are known as a trigger for GN such as HBV, HCV, HAV, CMV, EBV, parvovirus, or adenovirus. Then, we assessed HEV markers in those GN patients (n = 43) and healthy individuals (n = 36). Characteristics of GN patients and healthy subjects are presented in Table 2. The mean age for GN patients and healthy controls was 61 and 60 years, respectively. GN patients had elevated serum urea (mean 78.5 mg/dL) and higher serum creatinine (mean 2.2 mg/dL) but a reduced glomerular filtration rate (GFR) (mean 36 mL/min/1.73 m^2^) compared to healthy subjects who had the following mean values: 22.7 mg/dL, 0.84 mg/dL, and 92 mL/min/1.73 m^2^ for serum urea, serum creatinine, and GFR, respectively. Regarding the liver function tests, GN patients and healthy subjects had a normal level of alanine transaminase (mean ALT values 22.4 IU/L vs. 19.6 IU/L in GN and healthy controls, respectively), aspartate transaminase (AST mean 27.8 IU/L vs. 23.3 IU/L in GN and healthy controls, respectively), and bilirubin levels (mean total bilirubin values 0.72 mg/dL vs. 0.61 mg/dL in GN and healthy controls, respectively). While GN patients had a significantly lower level of serum albumin (mean 3.7 g/dL) compared to healthy controls (mean 4.27 g/dL). There were no significant differences between both groups in terms of gender, age, liver transaminase, and bilirubin. None of the GN patients nor healthy subjects tested positive for anti-HEV IgM or HEV RNA. In GN patients, 26 out of 43 patients (60.5%) were positive for anti-HEV IgG, while 9 out of 36 (25%) healthy subjects were IgG-positive. The seroprevalence of anti-HEV IgG is significantly higher in GN patients than in healthy individuals (Table 2).

### 3.2. Comparison between HEV-Seropositive and HEV-Seronegative GN Patients and Healthy Controls

**Figure 2:**
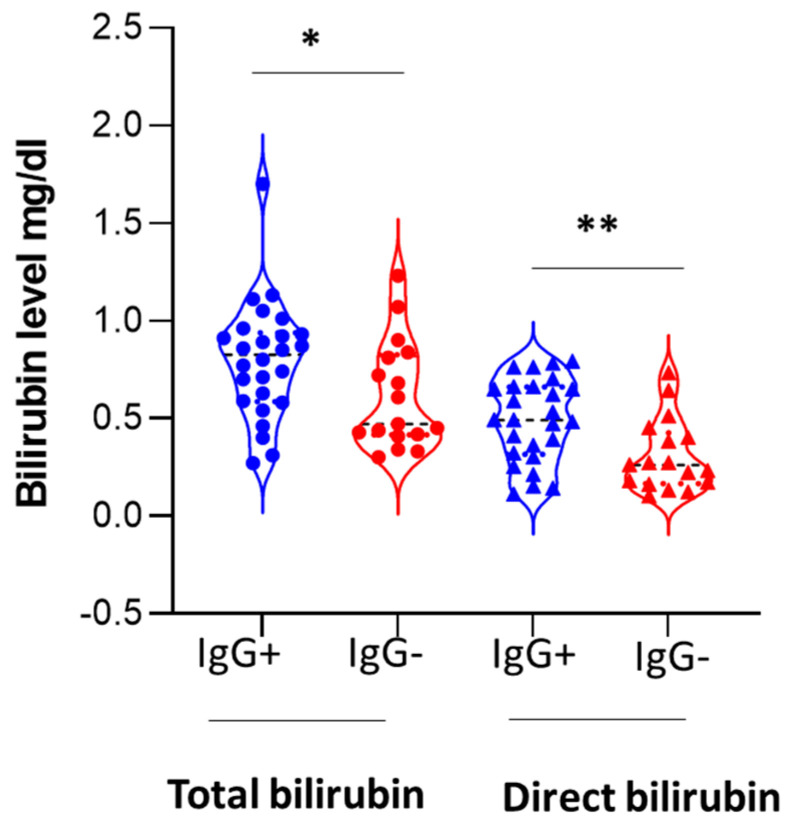
Bilirubin level in HEV seropositive and seronegative GN patients. Violin plot shows higher levels of total bilirubin and direct bilirubin in IgG+GN patients compared to IgG−GN patients. Blue represents seropositive GN patients. Red represents seronegative GN patients. *, ** represented *p* < 0.05 and 0.01, respectively, as determined by the Mann–Whitney test.

We compared anti-HEV IgG-positive GN patients versus anti-HEV IgG-negative GN patients. The mean ages of seropositive and seronegative GN patients were comparable (Table 3). The serum levels of urea (79 mg/dL in the seronegative group vs. 78.2 mg/dl in the seropositive group) and serum creatinine (2.15 mg/dL and 2.25 mg/dL in seronegative and seropositive GN patients, respectively) were comparable in both groups (Table 3). There was no difference in the estimated GFR between HEV-seronegative GN and HEV-seropositive GN patients. Regarding the liver function tests, the levels of ALT, AST, and albumin were similar in both groups (Table 3). The ALT level was within the normal range at around 22 IU/L in both groups. Similarly, the AST value was within the normal range, and the mean value was 29 IU/L in seronegative GN patients and 27 IU/mL in seropositive GN patients. There were no significant differences between both groups in terms of age, sex, kidney function tests, serum albumin, and liver transaminase levels (Table 3). Seropositive GN patients had a significantly higher total bilirubin level (mean 0.8 mg/dL) compared to seronegative GN patients (mean 0.6 mg/dL) and there was a significant increase in direct bilirubin levels in HEV-seropositive GN patients than in HEV-seronegative patients (Figure 2, Table 3).

In healthy subjects, 9 out of 36 subjects were positive for anti-HEV IgG. We compared the demographic and laboratory parameters in HEV-seropositive and HEV-seronegative healthy individuals (Table 4). There was no difference between the two groups in terms of age and gender. Additionally, the kidney function parameters (serum creatinine, serum urea, and estimated glomerular filtration rate) were comparable in both groups (Table 4). Similarly, the liver function tests (liver transaminases, bilirubin, and serum albumin) were not different between HEV-seropositive and HEV-seronegative healthy individuals (Table 4).

### 3.3. Liver Function Tests in HEV-Seropositive GN Patients and HEV-Seropositive Healthy Subjects

**Figure 3:**
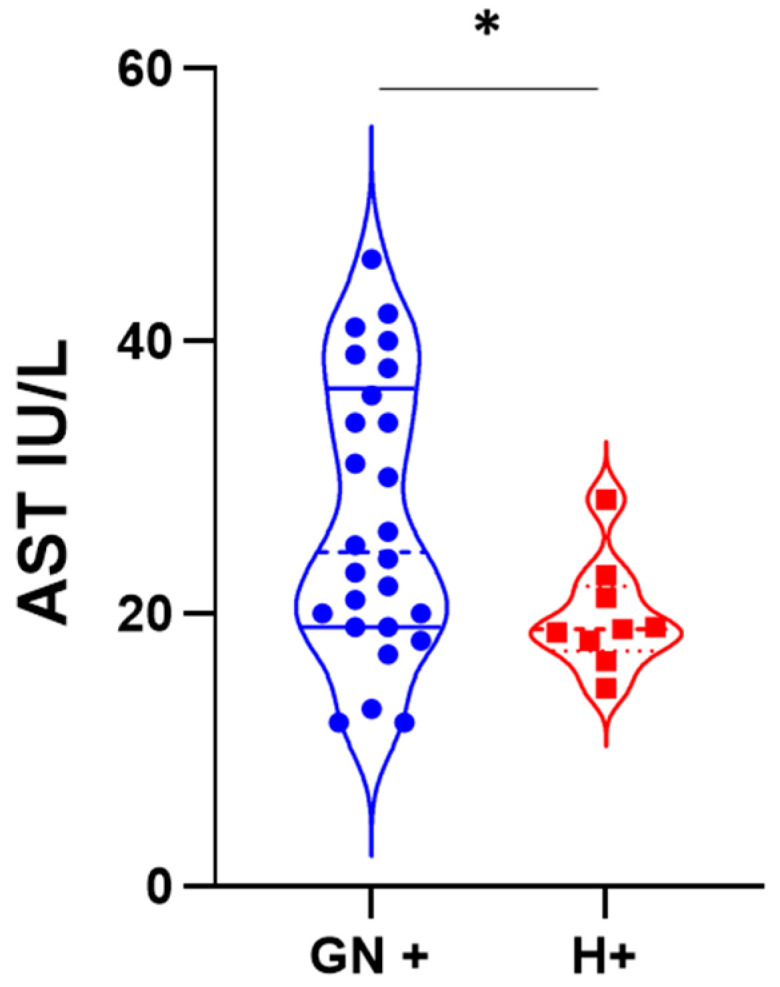
AST value in HEV seropositive subjects. AST level was compared between HEV-seropositive GN patients (blue circles, abbreviated as GN+) and HEV-seropositive healthy individuals (red squares, abbreviated as H+). * Indicated that *p* < 0.05 as determined by the Mann–Whitney test or Student’s *t*-test.

We compared the demographic and laboratory parameters between HEV-seropositive GN patients and HEV-seropositive healthy subjects (Table 3 and Table 4). There were no differences between either group in terms of age or gender. Regarding liver transaminases, ALT values were matched in both groups. However, AST values were significantly higher compared to HEV seropositive healthy subjects (Figure 3). HEV-seropositive GN patients had higher bilirubin but lower albumin compared to HEV-seropositive healthy subjects (Table 2 and Table 3); however, the results were not statistically significant.

## 4. Discussion

GN levels were recorded in HEV-infected patients [3]. However, the causal association between GN development and HEV infection is not confirmed. Membranoproliferative glomerulonephritis was developed in two solid organ transplants after getting an infection with HEV [9]. In a parallel line, Choi and colleagues reported that de novo glomerulonephritis was developed in four renal transplants, and two out of four patients were linked to HEV infection [34]. These two previous reports included French and German transplant recipients, respectively [9,34]. The common features of GN development in the previous studies were immunosuppressed patients, chronic HEV infections, and genotype 3 which is commonly circulating in these regions [9,34]. However, Guinault et al. reported that HEV infection could cause cryoglobulinemic glomerulonephritis in immunocompetent patients [5]. Most of the reported cases of HEV-associated GN were combined with cryoglobulinemia [5,6,9]. Similarly, cryoglobulinemic glomerulonephritis was also recorded in HCV-infected patients [35]. HEV infection could be an independent predicator for cryoglobulinemia, especially in solid organ transplant patients [36]. Interestingly, successful treatment with ribavirin improved kidney functions and resolved these complications [34,36,37]. Furthermore, Taton et al. reported that HEV infection was the causative agent of de novo membranous nephropathy that developed in a patient who underwent kidney transplantation [38]. On the other hand, Pischke and colleagues reported that past exposure to HEV-3 infection in German patients was not linked to the development of GN in general but could link to membranoproliferative glomerulonephritis [11].

This study included GN patients enrolled in Egyptian hospitals. In Egypt, HEV-1 is endemic [22,27,28,29] and HEV-3 is less predominant [29,30]. A high prevalence of HEV-seropositive subjects was recorded in asymptomatic subjects [18]. Symptomatic HEV-infected cases were self-limiting diseases and, in some cases, progressed to fulminant liver failure [25,29]. A recent study showed that 5 patients out of 31 HEV-infected patients developed poor renal function during acute infection. Importantly, these patients were HEV-1 infected. HEV-infected patients with poor renal functions had lower albumin levels but higher ALT than patients with normal renal parameters [12]. Renal functions were improved in these patients alongside the improvement of liver functions and the disappearance of manifestations (after viral clearance) [12]. There were no further renal consequences recorded [12]. Although these data showed that HEV-1 could be associated with poor kidney performance, they did not show a link between HEV infections and GN development. The aim of this study is to assess HEV markers in GN patients and assess if HEV exposure could be a risk factor for GN development, especially in regions endemic to HEV-1 infections.

In this study, we assessed the prevalence of HEV among GN patients and age and sex-matched healthy controls. The prevalence of anti-HEV IgG (60.5%) was significantly higher in GN patients than in healthy controls (25%), suggesting that GN patients are a risk group for HEV exposure. HEV RNA and anti-HEV IgM were negative in GN patients. This is not surprising, since these patients did not have a clinical manifestation of acute hepatitis as shown by normal liver transaminases and bilirubin. These results suggest that exposure to HEV could be a risk factor for renal complications such as GN. On the other hand, Pischke and colleagues reported that the percentages of anti-HEV IgG among GN and healthy subjects were 22% and 29%, respectively, in regions endemic to HEV-3 [11]. Since there were no significant differences in HEV seropositivity between both groups, the authors hypothesized that there was no link between past HEV-3 exposure and GN development in general [11].

Several studies and/or case reports from European centers showed that GN, especially membranoproliferative glomerulonephritis, could be a complication of HEV infection [3]. Among French organ transplants (n = 51), 8 patients were diagnosed with HEV-3 infection and developed membranoproliferative glomerulonephritis and IgA nephropathy [9]. Additionally, among German renal transplant recipients (n = 1469), about 10% of patients had abnormal LFTs and 16 patients were diagnosed with chronic HEV infections; from whom two patients developed HEV-linked GN [34]. There were also other cases of GN development in kidney organ transplant recipients [39]. HEV-3 is endemic in Europe and most GN cases were associated with to the chronicity of infection that is a feature of HEV-3 infection but not HEV-1. However, few cases of HEV-1 infection were reported with GN development. Ali et al. described a case of membranous glomerulonephritis developed after HEV infection, and the genotype 1 was the most likely cause [13]. This patient tested negative for other viral hepatitis markers but positive for HEV markers. The patient developed proteinuria and analysis of renal biopsy revealed the deposition of IgG, IgA, and IgM antibodies with complement [13]. Additionally, Vikrant and Kumar described a case study of a 56-year-old male patient who developed severe hyperbilirubinemia and acute renal failure due to HEV infection [40]. HEV-1 was excreted in urine or showed renal manifestations in animal models such as cynomolgus monkeys and Mongolian gerbils following infection [7,41]. Monkeys infected with stool-derived HEV-1, excreted the virus in urine starting on day 10–15 post-infection and lasting up to day 25–28 post-infection [7]. HEV antigens persisted in the urine more than in the blood or stool, and HEV RNA persisted equally in these compartments [7]. HEV-infected monkeys showed abnormal histological lesions in the kidneys, but the serum urea, creatinine, and other KFTs were normal [7]. Mongolian gerbils infected with HEV-1 showed increased inflammatory responses and immune cell infiltration in the kidneys following the infection [41]. Collectively, the previous data showed that, though data on HEV-1 and renal disorders is scarce, this does not mean excluding or ignoring the ability of these strains to be associated with renal injury and/or GN.

Comparing anti-HEV IgG-positive GN patients versus anti-HEV-negative GN patients, we did not find any difference between the two groups in terms of age, gender, kidney function tests, liver transaminases, and albumin. Seropositive GN patients had higher bilirubin levels (total and direct bilirubin) compared to seronegative GN patients. Comparing HEV-seropositive healthy donors vs. HEV-seronegative healthy donors, we did not find any difference between the groups in terms of age, gender, liver transaminases, bilirubin, albumin, or KFTs. The finding that HEV-seropositive GN patients, but not HEV seropositive healthy individuals, had elevated bilirubin compared to the HEV-seronegative corresponding partner requires further assessment. Furthermore, HEV-seropositive GN patients had a significantly elevated AST level compared to HEV-seropositive healthy subjects. Probably the seropositive GN patients developed HEV infections during their life and this could explain these findings, but further studies should be performed to ascertain these points. Similarly, Pischke et al. showed that anti-HEV positive-GN patients had higher bilirubin levels compared to seronegative GN patients [11]. However, in the previous report, seropositive GN patients were older and had higher creatinine levels (reduced glomerular filtration rate) compared to seronegative GN patients [11]. The difference between the two studies could be due to differences in patient populations, geographical distributions, circulating viruses, etc.

This study has some limitations. First, this study showed a link between the anti-HEV IgG positivity and GN. We believe that to confirm the association between HEV-1 infection and GN development, we need to assess HEV markers in GN patients who had symptoms of acute hepatitis, i.e., during the acute stage of infection. However, since the extrahepatic disorders could be caused either by direct virus pathogenesis or indirectly through host–pathogen immunocomplexes [3], this type of cohort (GN patients who developed acute hepatitis symptoms) is limited.

Second, the number of patients included in this study is not large. However, we believe that this cohort (GN patients) from a region known for HEV-1 endemicity is a positive point. The findings of this study, as they apply generally to HEV-1 infection exposure or specifically to Egyptian GN patients, need further investigations. HCV-infected patients in Egypt also developed membranoproliferative glomerulonephritis. Electron microscope examination of the renal biopsies from these patients showed HCV particles [16]. Similarly, analysis of renal biopsies from HBV-infected children in Egypt revealed that HBV infection is a trigger for GN development in these children [17]. Third, absence of kidney biopsies and immunohistochemistry analysis is another limitation. Future studies should investigate HEV proteins and antigens in kidney biopsies from GN patients. Additionally, we have not tested for cryoglobulinemia in this cohort, which is associated with many HEV-associated GN cases [5,6,36,37].

Despite these limitations, we believe that this study provides evidence that previous exposure to HEV infections could be associated with the development of GN. Since the enrolled subjects were negative for HBV, HCV, adenovirus, CMV, and EBV markers, we believe that HEV exposure could be a trigger for GN development. In a parallel line, our previous study showed that abnormal kidney functions could develop during acute HEV infections [12]. Together, the two studies provide proof of the possible association between HEV (mainly genotype 1) and glomerular diseases. Importantly, HEV-1 is common in developing countries where the research on HEV is limited. Clinicians could benefit from this study by becoming aware of the possibility of the development of GN following HEV-1 infections and/or exposure.

## 5. Conclusions

Anti-HEV seropositivity was significantly higher in GN patients compared to healthy subjects. GN should be considered in patients with a history of HEV infections. Anti-HEV seropositive GN patients had elevated bilirubin levels compared to seronegative GN patients.

## Figures and Tables

**Table 1:** Primer sequences for detection other viruses in the study.

Virus	Primer Sequences	Ref.
Hepatitis A virus (HAV)	Forward primer HAV: 5′-ggtaggctacgggtgaaac-3′ Reverse primer HAV: 5′-aacaactcaccaatatccgc-3′ Probe HAV: 5′-CTTAGGCTAATACTTCTATGAAGAGATGC-3′ 5′ is labeled with fluorescent dye “FAM” and 3′ is labeled with fluorescent quencher TAMRA.	[31]
Adenovirus	Adeno primer 1: 5′-GCCACGGTGGGGTTTCTAAACTT-3′ Adeno primer 2: 5′-GCCCCAGTGGTCTTACATGCACATC-3′ Adenoprobe: 5′-TGCACCAGACCCGGGCTCAGGTACTCCGA-3′ 5′ is labeled with fluorescent dye “FAM” and 3′ is labeled with fluorescent quencher TAMRA.	[32]
Cytomegalovirus (CMV)	CMV primer 1: 5′-GGC AGC TAT CGT GAC TGG-3′ CMV primer 2: 5′-GAT CCG ACC CAT TGT CTA AG-3′	[33]

**Table 2:** Characteristics of GN patients and healthy donors enrolled in the study and assessment of HEV markers.

	GN Patients (n = 43)	Healthy Subjects (n = 36)	Statistics (*p* Value)
Age (years) (Median, Range (minimum–maximum))	65 (13–83)	60.5 (39–83)	ns
Sex (M/F)	22/21	23/13	ns
Serum urea (mg/dL) (Median, Range (minimum–maximum))	72 (29–205)	20 (15–41)	<0.0001 (S)
Serum creatinine (mg/dL) (Median, Range (minimum–maximum))	1.6 (1.2–5.9)	0.8 (0.6–1.2)	<0.0001 (S)
Estimated glomerular filtration rate (eGFR) mL/min/1.73 m^2^	39 (8–77)	91 (53–127)	<0.0001 (S)
Serum albumin g/dL	3.8 (2.01–5.00)	4.2 (3.5–5.2)	0.0167 *
Liver transaminases and bilirubin ALT (IU/L) AST (IU/L) Total bilirubin (mg/dL) Direct bilirubin (mg/dL)	21 (8–41) 28 (10–51) 0.7 (0.3–1.7) 0.4 (0.1–0.79)	18.9 (9.4–44.1) 19.9 (8.9–49.6) 0.6 (0.2–1) 0.3 (0.1–0.77)	ns ns ns ns
HEV markers HEV RNA Anti-HEV IgM Anti-HEV IgG	0/42 (0%) 0/42 (0%) 26/43 (60.5%)	0/36 (0%) 0/36 (0%) 9/36 (25%)	0.0029 **

All values are expressed as median (minimum–maximum). Normal values for kidney function tests: upper normal level values for serum urea are 45 mg/dL, and serum creatinine 1.3 mg/dL and 1.1 mg/dL in males and females, respectively. For liver function tests: the upper normal level values for liver transaminases (ALT) are 45 IU/L; and AST: 34 IU/L. The upper normal value for total bilirubin is 1.2 mg/dL, direct bilirubin: less than 0.3 mg/dL. The normal values of serum albumin: 3.5–5.2 g/dL. *p* > 0.05 is considered non-significant (ns). *, ** mean *p* values were less than 0.05 and 0.01, respectively.

**Table 3:** Characteristics of anti-HEV IgG positive and negative GN patients.

	HEV Seronegative GN Patients (n = 17)	HEV Seropositive GN Patients (n = 26)	Statistics (*p* Value)
Age (years) (mean ± SD)	60.7 ± 15.8	61.5 ± 16.8	ns
Sex (M/F)	7/10 (41.2/58.8%)	15/11 (57.7%/42.3%)	ns
Serum urea (mg/dL) (mean ± SD)	79 ± 35.1	78.2 ± 40.5	ns
Serum creatinine (mg/dL) (mean ± SD)	2.15 ± 1.0	2.25 ± 1.38	ns
Estimated glomerular filtration rate (eGFR) mL/min/1.73 m^2^	34.1 ± 12.9	37.8 ± 17.3	ns
Serum albumin g/dL	3.7 ± 1	3.7 ± 0.9	ns
Liver transaminases and bilirubin ALT (IU/L) AST (IU/L) Total bilirubin (mg/dl) Direct bilirubin (mg/dl)	22 ± 9.4 29 ± 12.2 0.6 ± 0.28 0.3 ± 0.19	22.7 ± 10 27 ± 10.1 0.8 ± 0.3 0.5 ± 0.21	ns ns 0.04 * 0.007 **

Values are represented as the mean ± standard deviation. The normal values of serum urea, serum creatinine, liver transaminases (ALT and AST), total bilirubin, direct bilirubin, and serum albumin are mentioned in the footnote of Table 2. ns: means are non-significant as *p* > 0.05 as determined by Mann–Whitney test. *, ** mean *p* values were less than 0.05 and 0.01, respectively.

**Table 4:** Characteristics of anti-HEV IgG positive and negative healthy subjects.

	HEV-Seronegative Healthy Subjects (*n* = 27)	HEV-Seropositive Healthy Subjects (*n* = 9)	Statistics (*p* Value)
Age (years) (mean ± SD)	60.2 ± 9.6	60.8 ± 12.10	ns
Sex (M/F)	19/8 (70.4/29.6%)	4/5 (44.4%/55.6%)	ns
Serum urea (mg/dL)	22.3 ± 7.37	23.8 ± 7.39	ns
Serum creatinine (mg/dL)	0.83 ± 0.15	0.86 ± 0.16	ns
Estimated glomerular filtration rate (eGFR) mL/min/1.73 m^2^	94 ± 17.2	86.7 ± 25.5	ns
Serum albumin g/dL	4.3 ± 0.49	4.1 ± 0.54	ns
Liver transaminases and bilirubin ALT (IU/L) AST (IU/L) Total bilirubin (mg/dL) Direct bilirubin (mg/dL)	19.4 ± 8.8 24.5 ± 11.4 0.6 ± 0.23 0.33 ± 0.21	20.1 ± 5.6 19.8 ± 4 0.7 ± 0.24 0.35 ± 0.19	ns ns ns ns

Values are represented as the mean ± standard deviation. The normal values of serum urea, serum creatinine, liver transaminases (ALT and AST), total bilirubin, direct bilirubin, and serum albumin are mentioned in the footnote of Table 2. ns: means are non-significant as *p* > 0.05 as determined by Mann–Whitney test or *t*-test.

## Data Availability

The study data are present in the main text, and for further inquiries please contact the corresponding authors.
